# Adult Caretaker Engagement and School Connectedness and Association with Substance Use, Indicators of Emotional Well-Being and Suicide Risk, and Experiences with Violence Among American Indian or Alaska Native High School Students — Youth Risk Behavior Survey, United States, 2023

**DOI:** 10.15585/mmwr.su7304a2

**Published:** 2024-10-10

**Authors:** Sherry Everett Jones, Delight E. Satter, Julianna Reece, Jessica A. Larson, Laura M. Mercer Kollar, Phyllis Holditch Niolon, Laima Licitis, Jonetta J. Mpofu, Lisa Whittle, Trevor W. Newby, Jemekia E. Thornton, Lindsay Trujillo, Kathleen A. Ethier

**Affiliations:** ^1^Division of Adolescent and School Health, National Center for Chronic Disease Prevention and Health Promotion, CDC, Atlanta, Georgia; ^2^National Center for State, Tribal, Local, and Territorial Public Health Infrastructure and Workforce, CDC, Atlanta, Georgia, and Elder and Tribal member, Confederated Tribes of Grand Ronde; ^3^Division of Population Health National Center for Chronic Disease Prevention and Health Promotion, CDC, Atlanta, Georgia, and Navajo/Dine’; ^4^Division of Behavioral Health, Office of Clinical and Preventive Services, Indian Health Service, Rockville, Maryland, and Organized Village of Kake; ^5^Division of Overdose Prevention, National Center for Injury Prevention and Control, CDC, Atlanta, Georgia; ^6^Division of Violence Prevention, National Center for Injury Prevention and Control, CDC, Atlanta, Georgia; ^7^Oak Ridge Institute for Science and Education, Oak Ridge, Tennessee; ^8^U.S. Public Health Service Commissioned Corps, Rockville, Maryland

## Abstract

The strength of American Indian and Alaska Native (AI/AN) communities comes from generations of Indigenous traditions, language, culture, and knowledge. These strengths have been challenged by a complex set of systemic, structural, and social factors related to historical and intergenerational trauma that affects the health of AI/AN communities. Furthermore, AI/AN population health data often are inaccurate because of analytic coding practices that do not account for multiracial and ethnic AI/AN identification and inadequate because of statistical suppression. The 2023 national Youth Risk Behavior Survey included a supplemental sample of AI/AN high school students. Coding of race and ethnicity was inclusive of all AI/AN students, even if they also identified as another race or as Hispanic or Latino, providing comprehensive data on health behaviors and experiences among AI/AN high school students nationwide. Adult caretaker engagement and school connectedness and their association with 13 health behaviors and experiences were examined, including five types of current substance use, four indicators of emotional well-being and suicide risk, and four types of violence. Pairwise *t*-tests and adjusted prevalence ratios from logistic regression models identified significant associations between exposure and outcome variables. Among AI/AN students, having an adult who always tried to meet their basic needs, high parental monitoring, and high school connectedness were associated with lower prevalence of certain measures of substance use, poor emotional well-being and suicide risk, and violence. Compared with non-AI/AN students, the prevalence of current electronic vapor product use, current marijuana use, attempted suicide, and experience of sexual violence was higher among AI/AN students.

This report presents the most comprehensive, up-to-date data on substance use, indicators of emotional well-being and suicide risk, and experiences with violence among AI/AN high school students nationwide. The findings suggest the importance of engaged household adults and school connectedness in promoting emotional well-being and preventing substance use, suicide-related behavior, and experiences of violence among AI/AN students. Understanding the historical context and incorporating Indigenous knowledge when developing interventions focused on AI/AN youths are critical to ensure such interventions are successful in improving AI/AN health and well-being.

## Introduction

The strength of American Indian and Alaska Native (AI/AN) communities comes from generations of Indigenous traditions, language, culture, and knowledge (*1*–*4*). These strengths have, at times, been lost or challenged by a complex set of systemic, structural, and social factors tied to historical and intergenerational trauma (*5*) resulting from the U.S. government’s ethnocidal (e.g., Federal assimilation, termination, and relocation) and genocidal policies (e.g., military actions and forced relocation) (*4*). Health disparities that emerge because of these factors are best addressed with interventions that leverage the strengths of AI/AN communities (*1*). Growing up in a safe and stable environment, including one with nurturing relationships with caretakers (i.e., parents, other family members, or other adults in the community) who make sure basic needs are met, can reduce or prevent childhood and adolescent risk behaviors and trauma (*6*,*7*). Such nurturing relationships include boundary setting and monitoring children’s activities, location, and companions (*8*). Similarly, school connectedness (i.e., students feel close to persons at their school and believe that adults and peers at school care about them, their well-being, and their success) as well as parent or other caretaker engagement have been demonstrated to be particularly protective against behaviors and experiences related to mental health, substance use, sexual activity, and violence among students overall (*8*,*9*). The findings in this report address the need for more research that examines the association between such protective factors and substance use, emotional well-being and suicide risk, and experiences with violence among AI/AN youths.

Data are critical to public health decision-making and priority setting; however, for AI/AN persons, population-based health data are often low quality or inaccurate because of analytic coding practices that do not account for multiracial and ethnic AI/AN identification, lack of inclusion in study samples, and small sample sizes that result in low precision or statistical suppression (*2*,*10*,*11*). For example, one study found that typical analytic strategies used to code race and ethnicity (i.e., counting respondents as AI/AN only if they identified as single race AI/AN) represented only 18% of all AI/AN high school students (*11*). Most (82%) of those who identify as AI/AN also identify as Hispanic or Latino (Hispanic), or as having more than one racial identity (*11*). Using both a supplemental sample of AI/AN students and an inclusive method for coding AI/AN race, this report represents the most comprehensive, up-to-date data on health behaviors and experiences among AI/AN high school students in grades 9–12 nationwide.

The findings provided in this report will be useful for public health and education practitioners working with tribal and urban Indian communities for at least two reasons. First, these data provide the most comprehensive, up-to-date data on substance use, emotional well-being and suicide risk, and experiences with violence among AI/AN high school students nationwide. Second, these data underscore the importance of adult caretaking, parental monitoring, and school connectedness for AI/AN youths’ health and well-being. Understanding the historical context and incorporating Indigenous knowledge when developing interventions focused on AI/AN youths are critical to ensure such interventions are successful in improving AI/AN health and well-being.

## Methods

### Data Source

This report includes data from the 2023 YRBS (N = 20,103), a cross-sectional, school-based survey conducted biennially since 1991. Each survey year, CDC collects data from a nationally representative sample of public and private school students in grades 9–12 in the 50 U.S. states and the District of Columbia. Additional information about YRBS sampling, data collection, response rates, and processing is available in the overview report of this supplement (*12*). The prevalence estimates for adult caretaker engagement and school connectedness for the study population overall and stratified by sex, race and ethnicity, grade, and sexual identity are available at https://nccd.cdc.gov/youthonline/App/Default.aspx. The full YRBS questionnaire, data sets, and documentation are available at https://www.cdc.gov/yrbs/index.html. Institutional review boards at CDC and ICF, the survey contractor, approved the protocol for YRBS. Data collection was conducted consistent with applicable Federal law and CDC policy.*

### Measures

The main exposure variables of interest were household adult caretaking, parental monitoring, and school connectedness (Table 1). One question asked how often during the student’s lifetime there had been an adult in the student’s household who tried to make sure their basic needs were met. Another asked how often the student’s parents or other adults in the family knew where the student was going and with whom they would be (i.e., parental monitoring). The last question asked how close they felt to persons at school (i.e., school connectedness). Thirteen health behaviors and experiences were used as outcome variables, including five types of current substance use, four indicators of emotional well-being and suicide risk, and four types of violence.

**TABLE 1:** Questions, response options, and analytic coding for adult caretaking, school connectedness, substance use, indicators of emotional well-being and suicide risk, and experiences with violence among American Indian or Alaska Native high school students — Youth Risk Behavior Survey, United States, 2023

Variable	Question	Response option	Analytic coding
**Adult caretaking**			
Household adult tried to meet their basic needs	During your life, how often has there been an adult in your household who tried hard to make sure your basic needs were met, such as looking after your safety and making sure you had clean clothes and enough to eat?	Never, rarely, sometimes, most of the time, or always	Always versus not always (never, rarely, sometimes, or most of the time)
Parental monitoring	How often do your parents or other adults in your family know where you are going or with whom you will be?	Never, rarely, sometimes, most of the time, or always	High (most of the time, always) versus low (never, rarely, or sometimes)
**School connectedness**			
School connectedness	Do you agree or disagree that you feel close to people at your school?	Strongly agree, agree, not sure, disagree, or strongly disagree	High (strongly agree or agree) versus low (not sure, disagree, or strongly disagree)
**Substance use**			
Current cigarette use	During the past 30 days, on how many days did you smoke cigarettes? [note: the question did not distinguish between commercial and ceremonial use of tobacco]	0 days, 1 or 2 days, 3–5 days, 6–9 days, 10–19 days, 20–29 days, or all 30 days	Yes (≥1 day) versus no (0 days)
Current electronic vapor product use	During the past 30 days, on how many days did you use an electronic vapor product? [note: the question did not distinguish between commercial and ceremonial use of tobacco]	0 days, 1 or 2 days, 3–5 days, 6–9 days, 10–19 days, 20–29 days, or all 30 days	Yes (≥1 day) versus no (0 days)
Current marijuana use	During the past 30 days, how many times did you use marijuana?	0 times, 1 or 2 times, 3–9 times, 10–19 times, 20–39 times, or ≥40 times	Yes (≥1 time) versus no (0 times)
Current alcohol use	During the past 30 days, on how many days did you have at least one drink of alcohol?	0 days, 1 or 2 days, 3–5 days, 6–9 days, 10–19 days, 20–29 days, or all 30 days	Yes (≥1 day) versus no (0 days)
Current prescription opioid misuse	During the past 30 days, how many times did you take prescription pain medicine without a doctor's prescription or differently than how a doctor told you to use it?	0 times, 1 or 2 times, 3–9 times, 10–19 times, 20–39 times, or ≥40 times	Yes (≥1 time) versus no (0 times)
**Indicator of emotional well-being and suicide risk**			
Persistent feelings of sadness or hopelessness	During the past 12 months, did you ever feel so sad or hopeless almost every day for two weeks or more in a row that you stopped doing some usual activities?	Yes or no	Yes versus no
Poor mental health	During the past 30 days, how often was your mental health not good? (Poor mental health includes stress, anxiety, and depression.)	Never, rarely, sometimes, most of the time, or always	Yes (most of the time or always) versus no (never, rarely, or sometimes)
Seriously considered attempting suicide	During the past 12 months, did you ever seriously consider attempting suicide?	Yes or no	Yes versus no
Attempted suicide	During the past 12 months, how many times did you actually attempt suicide?	0 times, 1 time, 2 or 3 times, 4 or 5 times, or ≥6 times	Yes (≥1 time) versus no (0 times)
**Experience with violence**			
Ever physically forced to have sexual intercourse	Have you ever been physically forced to have sexual intercourse when you did not want to?	Yes or no	Yes versus no
Sexual violence victimization by anyone	During the past 12 months, how many times did anyone force you to do sexual things that you did not want to do? (Count such things as kissing, touching, or being physically forced to have sexual intercourse.)	0 times, 1 time, 2 or 3 times, 4 or 5 times, or ≥6 times	Yes (≥1 time) versus no (0 times)
Bullied on school property	During the past 12 months, have you ever been bullied on school property?	Yes or no	Yes versus no
Electronically bullied	During the past 12 months, have you ever been electronically bullied? (Count being bullied through texting, Instagram, Facebook, or other social media.)	Yes or no	Yes versus no

Demographic characteristics used in this study included sex (female or male), grade (9, 10, 11, or 12) and race and ethnicity. Students were asked their race and ethnicity using two questions. First, students were asked, “Are you Hispanic or Latino?” (yes or no). Second, students were asked, “What is your race? (Select one or more responses)” (American Indian or Alaska Native [AI/AN], Asian, Black or African American [Black], Native Hawaiian or other Pacific Islander [NH/OPI], or White). Consistent with recommendations for coding race and ethnicity when analyzing surveillance data with a focus on the AI/AN population (*11*), AI/AN race and ethnicity were coded to be inclusive of students with any mention of AI/AN (i.e., single race, multiracial, and Hispanic or Latino [Hispanic] AI/AN), with two exceptions. First, students who chose all five race categories were not included in the analytic sample because of concerns of interpretation and data quality, and second, students must have responded to both the race and ethnicity questions. All non-AI/AN students served as the comparison group.

### Analysis

For each behavior and experience, weighted prevalence estimates and 95% CIs were calculated overall (i.e., the national sample with all races and ethnicities combined) (N = 20,103) and then among AI/AN (N = 2,770) and non-AI/AN (N = 15,699) students. This study compared differences in the prevalence of behaviors and experiences between AI/AN and non-AI/AN students using pairwise *t*-tests. All prevalence estimates and measures of association used Taylor series linearization. Tests were considered statistically significant at the p<0.05 level. Among AI/AN students, adjusted prevalence ratios were calculated using logistic regression with predicted marginals, which controlled for sex and grade, to examine the association between household adult caretaking, parental monitoring, and school connectedness and the 13 outcome variables. Adjusted prevalence ratios (aPR) were considered statistically significant if the 95% CIs did not include 1.0. All analyses were conducted using SAS-callable SUDAAN (version 11.0.4; RTI International) to account for the complex sampling design and weighting.

## Results

### Household Adult Caretaking, Parental Monitoring, and School Connectedness

Overall, 74.7% of students had an adult in the household who always tried to meet their basic needs of safety, clothing, and food; 84.0% of students had a parent or other adult in the family who most of the time or always knew where they were going and with whom they will be (i.e., parental monitoring); and 55.3% felt close to persons at school (i.e., school connectedness) (Table 2). The prevalence of having a household adult who always tried to meet their basic needs was lower among AI/AN students (67.7%) compared with non-AI/AN students (75.3%).

**TABLE 2:** Prevalences of adult caretaking, school connectedness, substance use, indicators of emotional well-being and suicide risk, and experiences with violence* among high school students, overall^†^ and by American Indian or Alaska Native identity^§^ — Youth Risk Behavior Survey, United States, 2023

Variable	Students overall	AI/AN students	Non-AI/AN students
	% (95% CI)	% (95% CI)	% (95% CI)
**Adult caretaking**			
Household adult tried to meet their basic needs (always)	74.7 (72.1–77.1)	67.7 (60.5–74.1)	75.3 (72.8–77.6)^¶^
Parental monitoring (high)	84.0 (81.2–86.5)	74.7 (61.9–84.4)	84.6 (82.5–86.6)
**School connectedness**			
School connectedness (high)	55.3 (52.8–57.8)	51.5 (46.4–56.5)	56.1 (53.4–58.9)
**Substance use**			
Current cigarette use	3.5 (2.9–4.2)	3.9 (2.0–7.4)	3.6 (3.0–4.4)
Current electronic vapor product use	16.8 (15.4–18.2)	22.1 (17.5–27.4)	17.0 (15.6–18.6)^¶^
Current alcohol use	22.1 (20.5–23.8)	26.1 (20.6–32.5)	22.5 (20.9–24.3)
Current marijuana use	17.0 (15.4–18.7)	23.0 (18.4–28.4)	17.3 (15.7–19.0)^¶^
Current prescription opioid misuse	4.4 (3.9–5.0)	4.5 (3.0–6.8)	4.3 (3.7–5.0)
**Indicator of emotional well-being and suicide risk**			
Persistent feelings of sadness or hopelessness	39.7 (37.7–41.7)	45.1 (37.6–52.9)	39.5 (37.4–41.5)
Poor mental health	28.5 (26.7–30.4)	30.4 (23.7–38.1)	29.2 (27.5–31.0)
Seriously considered attempting suicide	20.4 (18.7–22.3)	20.9 (15.6–27.3)	20.8 (19.0–22.7)
Attempted suicide	9.5 (8.4–10.7)	14.4 (9.9–20.6)	9.4 (8.3–10.5)^¶^
**Experience with violence**			
Ever physically forced to have sexual intercourse	8.6 (7.7–9.6)	13.4 (9.8–18.0)	8.6 (7.6–9.7)^¶^
Sexual violence victimization by anyone	11.4 (10.4–12.4)	14.1 (9.8–19.9)	11.4 (10.4–12.6)
Bullied on school property	19.2 (17.3–21.4)	20.3 (14.9–27.0)	19.8 (17.8–21.9)
Electronically bullied	16.3 (14.2–18.5)	19.1 (14.8–24.2)	16.9 (14.8–19.1)

**Abbreviation:** AI/AN = American Indian or Alaska Native.

* Refer to Table 1 for variable definitions.

^†^ N = 20,103 respondents. The total number of students answering each question varied. Data might be missing because 1) the question did not appear in that student’s questionnaire, 2) the student did not answer the question, or 3) the response was set to missing because of an out-of-range response or logical inconsistency. Percentages in each category are calculated on the known data.

^§^ The coding of race and ethnicity was inclusive of all students who identified as AI/AN, even if they also identified as another race or as Hispanic or Latino (Hispanic). AI/AN: N = 2,770 respondents; non-AI/AN: N = 15,699 respondents.

^¶^ Significantly different from AI/AN based on *t*-test analysis with Taylor series linearization (p<0.05).

### Substance Use

Overall, the prevalence of current cigarette use was 3.5%, current electronic vapor product use was 16.8%, current alcohol use was 22.1%, current marijuana use was 17.0%, and current prescription opioid misuse was 4.4%. The prevalence of current electronic vapor product use and current marijuana use was higher among AI/AN students (22.1% and 23.0%, respectively) compared with non-AI/AN students (17.0% and 17.3%, respectively).

Among AI/AN students, having an adult in the household who always tried to meet their basic needs, compared with not always, was associated with lower prevalence of current electronic vapor product use (16.8% versus 27.8%; aPR = 0.58) (Tables 3 and 4) (Figure). High parental monitoring, compared with low parental monitoring, was associated with lower prevalence of current cigarette use (0.9% versus 11.4%; aPR = 0.07), current electronic vapor product use (15.5% versus 34.8%; aPR = 0.44), and current prescription opioid misuse (3.1% versus 11.6%; aPR = 0.28). High school connectedness, compared with low school connectedness, was associated with lower prevalence of current electronic vapor product use (15.9% versus 27.2%; aPR = 0.53).

**TABLE 3:** Prevalence of substance use, indicators of emotional well-being and suicide risk, and experiences with violence among American Indian or Alaska Native high school students,* by household adult tried to meet their basic needs, parental monitoring, and school connectedness^†^— Youth Risk Behavior Survey, United States, 2023

Variable	Household adult tried to meet their basic needs^§^		Parental monitoring^¶^		School connectedness**	
	Always	Not always	High	Low	High	Low
	% (95% CI)	% (95% CI)	% (95% CI)	% (95% CI)	% (95% CI)	% (95% CI)
**Substance use**						
Current cigarette use	2.1 (0.8–5.5)	5.1 (3.1–8.3)	0.9 (0.5–1.6)	11.4 (6.3–19.8)	3.4 (1.0–11.3)	3.9 (2.0–7.5)
Current electronic vapor product use	16.8 (12.3–22.6)	27.8 (19.6–37.8)	15.5 (12.0–19.8)	34.8 (23.5–48.0)	15.9 (9.2–26.3)	27.2 (21.7–33.5)
Current alcohol use	22.4 (17.0–28.8)	28.6 (18.7–41.1)	23.1 (17.9–29.4)	34.1 (20.8–50.5)	25.1 (18.1–33.8)	27.1 (18.9–37.3)
Current marijuana use	21.4 (16.1–27.9)	26.2 (17.2–37.6)	21.9 (17.1–27.6)	26.7 (17.3–38.7)	20.9 (15.0–28.3)	27.9 (20.8–36.3)
Current prescription opioid misuse	3.8 (1.9–7.4)	6.2 (3.5–10.9)	3.1 (1.6–5.9)	11.6 (6.1–20.8)	4.2 (1.7–9.9)	5.4 (3.3–8.8)
**Indicator of emotional well-being and suicide risk**						
Persistent feelings of sadness or hopelessness	40.7 (32.3–49.7)	54.2 (44.0–64.0)	41.7 (33.7–50.1)	52.3 (42.3–62.1)	36.0 (28.2–44.7)	57.1 (45.9–67.6)
Poor mental health	30.9 (24.2–38.4)	36.1 (23.1–51.6)	30.8 (24.0–38.6)	22.2 (11.7–38.0)	23.0 (15.3–33.1)	38.9 (30.3–48.2)
Seriously considered attempting suicide	17.0 (11.0–25.2)	26.5 (17.7–37.7)	17.7 (12.1–25.2)	23.8 (13.9–37.6)	12.5 (8.1–18.8)	28.6 (19.8–39.4)
Attempted suicide	7.8 (4.2–14.0)	23.9 (14.2–37.4)	8.4 (4.6–14.6)	21.8 (15.3–30.2)	10.4 (5.3–19.5)	17.7 (11.9–25.6)
**Experience with violence**						
Ever physically forced to have sexual intercourse	11.1 (7.0–16.9)	18.6 (11.2–29.2)	13.7 (9.1–20.2)	15.1 (7.7–27.6)	10.8 (6.9–16.7)	18.1 (12.6–25.4)
Sexual violence victimization by anyone	9.3 (6.3–13.5)	18.9 (13.2–26.3)	12.5 (7.9–19.4)	21.5 (14.5–30.6)	10.2 (5.4–18.4)	18.7 (13.0–26.2)
Bullied on school property	18.6 (12.0–27.7)	25.6 (17.4–35.9)	20.1 (12.8–30.0)	19.2 (15.0–24.1)	17.0 (10.6–26.1)	25.1 (18.0–33.9)
Electronically bullied	13.5 (9.2–19.5)	32.3 (21.1–45.9)	15.9 (10.9–22.5)	21.7 (17.2–27.0)	13.0 (8.4–19.7)	24.9 (18.0–33.5)

* N = 2,770. The coding of race and ethnicity was inclusive of all students who identified as American Indian or Alaska Native, even if they also identified as another race or as Hispanic or Latino (Hispanic). The total number of American Indian or Alaska Native students answering each question varied. Data might be missing because 1) the question did not appear in that student’s questionnaire, 2) the student did not answer the question, or 3) the response was set to missing because of an out-of-range response or logical inconsistency. Percentages in each category are calculated on the known data.

^†^ Refer to Table 1 for variable definitions.

^§^ Always versus not always (never, rarely, sometimes, or most of the time).

^¶^ High (most of the time or always) versus low (never, rarely, or sometimes).

** High (strongly agree or agree) versus low (not sure, disagree, or strongly disagree).

**TABLE 4:** Adjusted prevalence ratios* for substance use, indicators of emotional well-being and suicide risk, and experiences with violence among American Indian or Alaska Native high school students,^†^ by household adult tried to meet their basic needs, parental monitoring, and school connectedness^§^ — Youth Risk Behavior Survey, United States, 2023

Variable	Household adult tried to meet their basic needs^¶^		Parental monitoring**		School connectedness^††^	
	Always	Not always	High	Low	High	Low
	aPR (95% CI)		aPR (95% CI)		aPR (95% CI)	
**Substance use**						
Current cigarette use	0.43 (0.14–1.35)	Ref	0.07 (0.04–0.14)^§§^	Ref	0.73 (0.20–2.73)	Ref
Current electronic vapor product use	0.58 (0.37–0.92)^§§^	Ref	0.44 (0.30–0.66)^§§^	Ref	0.53 (0.29–0.97)^§§^	Ref
Current alcohol use	0.77 (0.47–1.26)	Ref	0.71 (0.42–1.21)	Ref	0.94 (0.65–1.37)	Ref
Current marijuana use	0.79 (0.52–1.21)	Ref	0.85 (0.56–1.28)	Ref	0.77 (0.51–1.16)	Ref
Current prescription opioid misuse	0.49 (0.19–1.31)	Ref	0.28 (0.10–0.75)^§§^	Ref	0.56 (0.19–1.68)	Ref
**Indicator of emotional well-being and suicide risk**						
Persistent feelings of sadness or hopelessness	0.77 (0.61–0.98)^§§^	Ref	0.78 (0.59–1.03)	Ref	0.67 (0.56–0.80)^§§^	Ref
Poor mental health	0.86 (0.61–1.21)	Ref	1.40 (0.70–2.80)	Ref	0.61 (0.41–0.91)^§§^	Ref
Seriously considered attempting suicide	0.64 (0.41–1.00)^§§^	Ref	0.69 (0.42–1.12)	Ref	0.47 (0.32–0.70)^§§^	Ref
Attempted suicide	0.30 (0.14–0.61)^§§^	Ref	0.36 (0.20–0.66)^§§^	Ref	0.59 (0.33–1.07)	Ref
**Experience with violence**						
Ever physically forced to have sexual intercourse	0.54 (0.34–0.86)^§§^	Ref	0.85 (0.46–1.56)	Ref	0.68 (0.38–1.22)	Ref
Sexual violence victimization by anyone	0.46 (0.29–0.73)^§§^	Ref	0.51 (0.29–0.90)^§§^	Ref	0.59 (0.31–1.12)	Ref
Bullied on school property	0.79 (0.49–1.25)	Ref	1.02 (0.62–1.68)	Ref	0.71 (0.48–1.05)	Ref
Electronically bullied	0.45 (0.28–0.72)^§§^	Ref	0.69 (0.43–1.12)	Ref	0.59 (0.39–0.89)^§§^	Ref

**Abbreviations**: aPR = adjusted prevalence ratio; Ref = referent group.

* aPRs are based on logistic regression models adjusted for sex and grade.

^†^ N = 2,770. The coding of race and ethnicity was inclusive of all students who identified as American Indian or Alaska Native, even if they also identified as another race or as Hispanic or Latino (Hispanic). The total number of American Indian or Alaska Native students answering each question varied. Data might be missing because 1) the question did not appear in that student’s questionnaire, 2) the student did not answer the question, or 3) the response was set to missing because of an out-of-range response or logical inconsistency. Percentages in each category are calculated on the known data.

^§^ Refer to Table 1 for variable definitions.

^¶^ Always versus not always (never, rarely, sometimes, or most of the time).

** High (most of the time or always) versus low (never, rarely, or sometimes).

^††^ High (strongly agree or agree) versus low (not sure, disagree, or strongly disagree).

^§§^ aPRs were considered statistically significant; 95% CIs did not include 1.0 (p<0.05). Certain statistically significant aPRs have 95% CIs that include 1.0 because of rounding.

**FIGURE:**
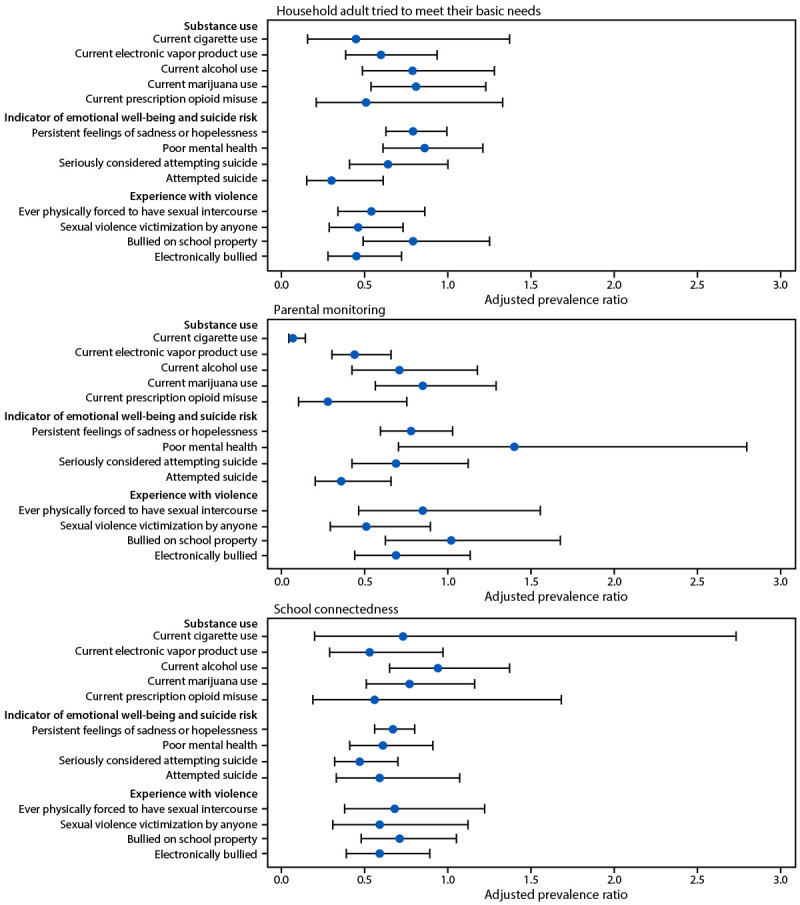
Adjusted prevalence ratios* for substance use, indicators of emotional well-being and suicide risk, and experiences with violence among American Indian or Alaska Native high school students, by household adult tried to meet their basic needs, parental monitoring, and school connectedness — Youth Risk Behavior Survey, United States, 2023 * aPRs are based on logistic regression models adjusted for sex and grade. Bars indicate 95% CIs.

### Indicators of Emotional Well-Being and Suicide Risk

Overall, 39.7% of students experienced persistent feelings of sadness or hopelessness, 28.5% had poor mental health, 20.4% had seriously considered attempting suicide, and 9.5% had attempted suicide. The prevalence of attempted suicide was higher among AI/AN students (14.4%) compared with non-AI/AN students (9.4%).

Among AI/AN students, having an adult in the household who always tried to meet their basic needs, compared with not always, was associated with lower prevalence of persistent feelings of sadness or hopelessness (40.7% versus 54.2%; aPR = 0.77), having seriously considered attempting suicide (17.0% versus 26.5%; aPR = 0.64), and attempted suicide (7.8% versus 23.9%; aPR = 0.30). High parental monitoring, compared with low parental monitoring, was associated with lower prevalence of attempted suicide (8.4% versus 21.8%; aPR = 0.36). High school connectedness, compared with low school connectedness, was associated with lower prevalence of persistent feelings of sadness or hopelessness (36.0% versus 57.1%; aPR = 0.67), poor mental health (23.0% versus 38.9%; aPR = 0.61), and having seriously considered suicide (12.5% versus 28.6%; aPR = 0.47).

### Experiences with Violence

Overall, 8.6% of students had ever been physically forced to have sexual intercourse, 11.4% had experienced sexual violence victimization by anyone, 19.2% had been bullied on school property, and 16.3% had been electronically bullied. Having ever been physically forced to have sexual intercourse was higher among AI/AN students (13.4%) compared with non-AI/AN students (8.6%).

Among AI/AN students, having an adult in the household who always tried to meet their basic needs, compared with not always, was associated with lower prevalence of ever having been physically forced to have sexual intercourse (11.1% versus 18.6%; aPR = 0.54), sexual violence victimization by anyone (9.3% versus 18.9%; aPR = 0.46), and being electronically bullied (13.5% versus 32.3%; aPR = 0.45). High parental monitoring, compared with low parental monitoring, was associated with lower prevalence of sexual violence victimization by anyone (12.5% versus 21.5%; aPR = 0.51). High school connectedness, compared with low school connectedness, was associated with lower prevalence of being electronically bullied (13.0% versus 24.9%; aPR = 0.59).

## Discussion

Using a supplemental sample and an inclusive coding strategy to identify AI/AN students, 2023 YRBS data indicated that the prevalence of current electronic vapor product use, current marijuana use, attempted suicide, and ever having been physically forced to have sexual intercourse was higher among AI/AN students compared with non-AI/AN students. Among AI/AN students, the protective factors of household adult caretaking, parental monitoring, and school connectedness were associated with lower prevalence of certain measures of substance use, poor emotional well-being and suicide risk, and violence. The findings in this report are consistent with other findings that family engagement (*7*), parental monitoring (*9*), and school connectedness (*8*) were protective against risk behaviors among high school students.

This report’s findings can be used to guide interventions that address the unique needs of AI/AN communities. Traditional Indigenous knowledge has been embraced by the Federal government as a “valid form of evidence for inclusion in Federal policy, research and decision making” (*1*), and any interventions designed for tribal and urban Indian communities must “acknowledge historical context and past injustice” (*1*). Historical instances of systemic physical and intellectual separation of AI/AN populations from land, water, and social systems perpetuated the intentional loss of the AI/AN way of life, resulting in well-documented adverse health outcomes (*1*,*3*,*4*,*13*). However, AI/AN communities have long recognized the importance of Indigenous traditions, language, culture, and knowledge as protective factors that affect health and well-being of Indigenous youths, sometimes referred to as cultural, social, or collective resilience (*13*).

Findings from the 2019 National Indian Education Study (NIES), which considered AI/AN cultures and languages, demonstrated that Native language and culture programs supported student academic achievement, motivation, self-esteem, and pride in grades 4 and 8 (*14*). NIES also found that families involved in volunteer programs or a parent-teacher organization were more likely to have students who were academically high performing (*14*). Such work could be expanded to study the impacts of family engagement, school connectedness, and cultural resilience–focused programs on AI/AN high school students’ health behaviors and experiences. Findings from the 2023 YRBS indicated that two in three AI/AN students had a household adult who always tried to meet their basic needs during their lifetime and three in four students reported high parental monitoring, both of which were protective for various behaviors related to substance use, emotional well-being and suicide risk, and violence. Thus, caretaker engagement emerges as a critical component of cultural resilience–focused programs (*6*,*14*) and appears promising in reducing risk behaviors and experiences among AI/AN high school students.

CDC provides various resources for action, including Preventing Adverse Childhood Experiences (https://www.cdc.gov/violenceprevention/pdf/ACEs-Prevention-Resource_508.pdf), Suicide Prevention Resource for Action (https://www.cdc.gov/suicide/resources/prevention.html), and Promoting Mental Health and Well-being in Schools (https://www.cdc.gov/healthyyouth/mental-health-action-guide/index.html?s_cid=hy-2023). These resources offer strategies that are based on the best evidence to support young persons, including those who have experienced trauma. CDC’s What Works in Schools program (https://www.cdc.gov/healthyyouth/whatworks/index.htm) focuses on improving school connectedness and has been demonstrated to reduce behavioral risks and improve mental health and well-being. CDC is evaluating additional efforts to design culturally informed strategies that aim to serve the needs of AI/AN students.

## Limitations

General limitations for the YRBS are available in the overview report of this supplement (*12*). The findings in this report are subject to at least five additional limitations. First, the data in this report are cross-sectional and causality and temporality cannot be inferred despite significant associations. Second, the protective factors examined in this report are all complex constructs that might not be fully captured using one question. For example, parental monitoring involves a combination of factors related to caretaker communication and inquiry as well as child disclosure about where and with whom they will be (*9*). The school connectedness question does not differentiate relationships across peer groups, teachers, and staff (*8*). The question addressing having an adult in the household who tried to meet their basic needs does not fully describe the adult-child relationship (i.e., parent versus another caring adult) or the extent to which that adult was a consistent presence during childhood and into the teenage years. Third, Bureau of Indian Education–funded schools, because of their unique nature and location, often on tribally controlled lands, were not included in the sampling frame. The findings in this report are generalizable to AI/AN students attending public or private schools only. Fourth, YRBS data do not include information about any tribal affiliation, nor whether students lived on or off tribal lands. Residence in tribal lands (e.g., reservations or villages) could have implications for AI/AN youth health behaviors and experiences. Finally, although an advantage of this study is its inclusion of single race, multiracial, and Hispanic AI/AN students in the analysis, the findings should not be compared directly with those of other reports in which non-Hispanic single race AI/AN data are described.

## Future Directions

Despite study limitations, the 2023 YRBS contributes important information about AI/AN health behaviors and experiences and points to two future directions for public health data collection with AI/AN youths. First, the 2023 YRBS included a supplemental sample of AI/AN youths, and students were included in the analytic sample if they identified as AI/AN, even if they also identified as another race or as Hispanic. Coding strategies that account for AI/AN youths who are Hispanic and multiracial are critical to appropriately identifying AI/AN youths in public health data systems (*2*,*10*,*11*). Surveillance data that limit AI/AN data to a single race category, especially in many areas in which AI/AN populations are smaller in size relative to other racial or ethnic groups, can lead to misclassification, suppression of data, or loss of important data in a nondescript “other” or multiracial category (*2*,*10*). Quality surveillance data that reflect the multiplicity of AI/AN identity is necessary for future public health policy work.

Second, although national YRBS data are critical to support policy decisions at the national, tribal, state, territorial, or local school district levels, tribal nations also benefit from local and tribally representative data to support tribal policy decisions (*2*,*3*,*10*). Each of the 574 federally recognized tribes has unique cultures, traditions, resources, and needs (*1*,*3*). The Youth Risk Behavior Surveillance System (https://www.cdc.gov/yrbs/index.html) is designed to support tribal nation YRBSs and allows for the collection of YRBS data to support tribal public health and education decision-making while supporting tribal data sovereignty. As with states, school districts, and territories that collect YRBS data representative of their jurisdictions, tribal nations could use such data to describe risk behaviors, experiences, and protective factors; support health-related policies and legislation within the tribe or in local or state governments implementing policies and legislation that affect tribal members; plan and monitor programs; guide professional development; and seek funding (*15*). Meanwhile, outside of tribally coordinated YRBSs, other population studies and analysis of state and school district data could be conducted using the more inclusive race and ethnicity coding strategies used in this report and supported by other research (*10*,*11*). Those data could be used to guide tribally driven public health prevention and wellness programs that address individual and family risk and protective factors associated with the health of AI/AN youths.

## Conclusion

For the first time, the 2023 YRBS included a supplemental sample of AI/AN high school students. Furthermore, to best reflect AI/AN youths’ own racial identity, race and ethnicity were coded to include single race, multiracial, and Hispanic AI/AN students. Thus, the findings in this report are the most comprehensive, up-to-date data on risk behaviors and experiences among AI/AN high school students nationwide. Among AI/AN youths, lifetime presence of an adult who always tried to meet their basic needs, high parental monitoring, and high school connectedness were associated with lower prevalence of certain measures of substance use, indicators of emotional well-being and suicide risk, and violence. These findings, as with findings from studies examining other racial and ethnic groups (*7*–*9*), suggest the importance of engaged household adults and school connectedness in addressing substance use, suicide-related behavior, and violence among AI/AN students. Understanding the historical context and incorporating Indigenous knowledge (*1*) when developing interventions focused on AI/AN youths are critical to ensure such interventions are successful in improving AI/AN health and well-being.
